# Implementation of Aseptically Processed Human Placental Membrane Allografts Within a Comprehensive Sternal Wound Closure Strategy: A Three-Phase Experience

**DOI:** 10.3390/jcm14061877

**Published:** 2025-03-11

**Authors:** Zain Khalpey, Ujjawal Aditya Kumar, Pamela Hitscherich, Zacharya Khalpey, Tyler Phillips, Evangelia Chnari, Marc Long

**Affiliations:** 1 Department of Cardiac Surgery, HonorHealth, Suite 300, 10210 N 92nd St., Scottsdale, AZ 85258, USA; tylerphilly14@gmail.com; 2 Khalpey AI Lab, Applied & Translational AI Research Institute (ATARI), 10210 N 92nd St., Scottsdale, AZ 85258, USA; zach@khalpey.ai; 3 School of Clinical Medicine, University of Cambridge, Hills Road, Cambridge CB2 0SP, UK; 4 Department of Research & Development, MTF Biologics, Edison, NJ 08837, USA; pamela_hitscherich@mtf.org (P.H.); evangelia_chnari@mtf.org (E.C.); marc_long@mtf.org (M.L.)

**Keywords:** cardiac surgery, median sternotomy, open-heart surgery, sternal complications, placental allograft

## Abstract

**Background:** Sternal wound complications following median sternotomy result in poor outcomes. Novel approaches such as placental allografts are being explored to optimize wound closure. **Methods:** This study evaluated consecutive patients undergoing median sternotomy by a single surgeon as sternal closure strategies evolved. Initially, wires with autologous platelet-rich plasma (PRP) were used (Group 1). Subsequently, suture tapes with PRP and an aseptically processed amnion–chorion placental allograft (aACPA) were added (Group 2). Finally, PRP was discontinued (Group 3). Sternal infection, dehiscence, pain outcomes, hospital length of stay, and patient risk factors were analyzed. **Results:** Compared to Group 1, Groups 2 and 3 demonstrated significantly lower infection (0.7%, 0% vs. 9.3%, *p* = 0.0001) and dehiscence rates (0%, 0% vs. 8.7%, *p* < 0.0001). Significant postoperative pain at two weeks decreased from Group 1 to Groups 2 and 3 (18.7%, 4.7%, 3.1%, *p* < 0.0001), with similar improvements at one month (12.0%, 2.0%, 1.5%, *p* = 0.0005). Despite higher median risk factors in Group 3 than in Groups 1 and 2 (3 vs. 2, 2, *p* = 0.0305), a trend toward reduced hospital stay was observed (6 vs. 8, 7 days, *p* = 0.2298). **Conclusions:** Adding aACPA to sternal closure significantly reduced infections, dehiscence, and pain in high-risk cardiac surgery patients, with sustained benefits and no increase in operative times. These findings highlight aACPA’s potential to mitigate sternal complications, warranting further study in larger cohorts.

## 1. Introduction

Although minimally invasive approaches are becoming more common, the incision of choice in most cardiac surgeries is still the full median sternotomy, as it provides unrivaled access to and exposure of the heart and great vessels [1]. However, it carries significant risks, including sternal dehiscence and deep sternal wound infections, which lead to delayed recovery, prolonged inpatient stays, increased resource utilization, and poor prognosis. Sternal complications result in around 20% of 30-day hospital readmissions [2], highlighting their impact on patient outcomes and healthcare costs.

Patients who are obese or diabetic, those with chronic obstructive pulmonary disease (COPD), those who undergo pedicled or bilateral internal mammary artery use, those with prolonged cardiopulmonary bypass time, or those who undergo reoperation for bleeding postoperatively are at high risk of sternal complications [3,4]. Obesity particularly increases risk due to increased mechanical stress on the sternum, compromising reapproximation and wound healing [5]. The mechanical component of sternal closure is especially important in obese patients [6], as sternal wound dehiscence typically occurs on a shorter timescale than bone healing. COPD patients face similar challenges from frequent and forceful coughing. Smoking and diabetes-related microvascular changes further impair wound healing. Frailty, extremes of BMI, osteoporosis, and long-term steroid use also increase susceptibility to sternal instability and complications, with frail patients having twice the risk compared to their non-frail counterparts [7]. It is well established that extremes of BMI (below 18 kg/m^2^ or above 30 kg/m^2^) can confer a “frail phenotype” [8].

Traditional sternal closure with steel wires has been supplemented with various approaches to enhance healing. Platelet-rich plasma (PRP) emerged as an early adjunct, offering growth factors and cytokines that enhance soft tissue and bone healing [9]. However, even with prophylactic antibiotic treatment, vacuum-assisted closure (VAC) dressings, and antibiotic impregnation of the surgical site, our sternal complication rate remained relatively high. For high-risk patients, such as those with the risk factors already discussed, the sternal complication rate is known to be as high as 10% [10]. These challenges have driven the evolution of sternal closure techniques and the development of an enhanced wound protocol at our institution.

Traditional steel wires have been replaced with ultra-high-molecular-weight polyethylene suture tapes, which are stronger, distribute the load more effectively, and improve outcomes [11]. When combined with placental membranes and PRP, this approach offers mechanical and biological benefits. Regenerative medicine therapies, particularly aseptically processed amnion–chorion placental allografts (aACPAs), have garnered attention for their potential to augment wound closure and minimize complications [12]. These therapies have demonstrated promising efficacy in various surgical settings [13] and complex wound environments [14,15,16,17,18]. aACPAs have anti-inflammatory, antimicrobial, and pro-healing properties, thus promoting epithelialization and reducing scarring [19]. Retained extracellular matrix components may function as a scaffold for postoperative cell migration and proliferation, while the intrinsic cytokines and growth factors are suggested to provide a supportive environment for tissue remodeling [20].

This study evaluates the outcomes of our evolving institutional approach, which combines innovative closure systems and regenerative therapies to reduce sternal wound complications in high-risk cardiac surgery patients. Key metrics include intensive care unit (ICU) and hospital stay duration, infection and dehiscence rates, and postoperative pain.

In light of the escalating risk associated with cardiac surgical patients and the consequent demand for enhanced sternal closure techniques, this study endeavors to evaluate our implementation of an enhanced wound closure protocol across three phases and assess the feasibility of introducing a biologic therapy to support wound closure. The primary objective is to ascertain its potential to enhance patient outcomes and mitigate complications following median sternotomy.

## 2. Patients and Methods

This is a retrospective observational cross-sectional descriptive study of consecutive patients who underwent median sternotomy for cardiac surgery (isolated coronary artery bypass or valve surgery, combined coronary and valve surgery, or major aortic surgery) at our institution (HonorHealth, Scottsdale, AZ, USA) between January 2022 and September 2024. Any patients with previous sternal complications or a history of chronic opioid use, as well as those under 18 years of age, were excluded. In total, 23 patients were excluded. For prior sternal complications, 1, 3, and 2 patients were excluded from Groups 1, 2, and 3, respectively, and for chronic preoperative opioid use, 6, 7, and 4 patients were excluded from Groups 1, 2, and 3, respectively. The final sample size was 365 patients.

As part of implementing an enhanced wound healing protocol, the clinical practice evolved throughout the study period, transitioning from using steel sternal wires in April 2023 to discontinuing PRP use in May 2024. Patients in Group 1 were the last 150 patients who underwent surgery via sternotomy before this practice change, Group 2 patients consisted of the first 150 patients who underwent sternal closure using the new system (April 2023 to May 2024), and Group 3 comprised the first 65 patients in whom PRP use was discontinued. Institutional Review Board approval (IRB#23-0025, granted 26 April 2023) was obtained for outcome analysis. Informed consent was obtained from all patients for the relevant surgical procedures and anonymized study inclusion. All study methods were conducted following applicable guidelines and regulations for human subject work [21].

### 2.1. Data Collection

Demographic data, preoperative clinical characteristics, operative details, and data on postoperative outcomes were extracted from the institutional electronic health record system (Epic) [22], anonymized, and stored securely following information governance policies for health outcomes research data. Preoperative characteristics also included data on comorbidities that contribute to the risk of postoperative sternal complications. These included obesity (high body mass index/BMI), chronic obstructive pulmonary disease (COPD), diabetes mellitus (DM), prior sternotomy, smoking history, and long-term immunosuppressive medication use, such as steroids. Preoperative data also included key surgical risk scores (Society of Thoracic Surgeons/STS mortality, deep sternal wound infection). The STS mortality risk score is one of the two widely-used risk stratification scores in cardiac surgery, alongside the EuroSCORE 2. The internationally accepted American Heart Association (AHA) criteria for heart failure [23] and Kidney Disease: Improving Global Outcomes (KDIGO) criteria for chronic kidney disease [24] were used to define these two chronic conditions. Key operative times, including total cardiopulmonary bypass time and aortic cross-clamp/myocardial ischemia time, were recorded, as well as the total operative time. Postoperatively, outcomes recorded were hospital mortality, sternal wound infection, and sternal wound dehiscence. Sternal wound complications were defined using the STS criteria for entry into the National Adult Cardiac Surgical Database [25].

### 2.2. Operative Technique

Sternal reapproximation was the only stage at which the three groups in this study differed. Following sternal reapproximation, wound lavage was performed with a vancomycin solution in all 365 patients. Soft tissue and skin were closed with 0, 2–0, and 4–0 Stratafix sutures (Ethicon Inc., Cincinnati, OH, USA), and the skin was covered with a Prineo Dermabond dressing (Ethicon Inc.).

#### 2.2.1. Group 1: Steel Wire Closure with PRP

**Figure 1:**
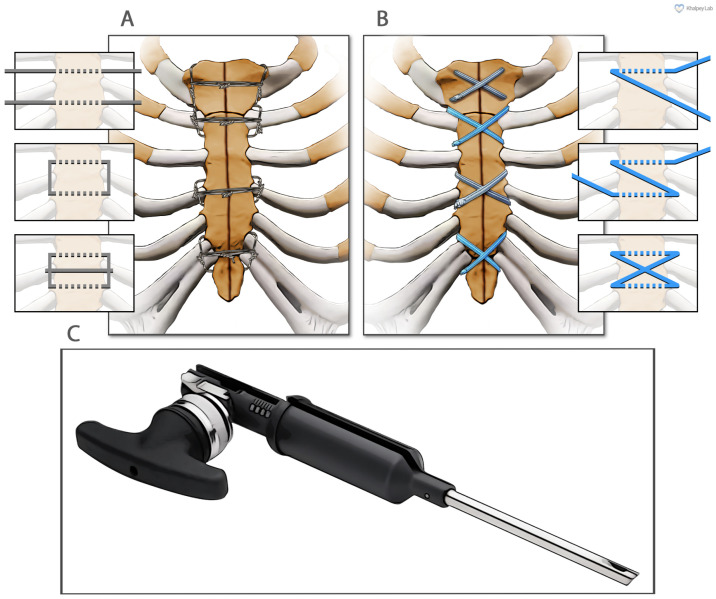
**The two different methods of sternal closure utilized in this study.** (**A**) Sternal wire sternal cerclage in a semi-Robicsek figure-of-eight pattern. (**B**) Suture tape sternal cerclage. (**C**) The suture tape tensioner device (Arthrex, Inc., Naples, FL, USA).

In these 150 patients, sternal reapproximation and closure were achieved using surgical steel wires in a semi-Robicsek figure-of-eight pattern (Figure 1A). PRP obtained from 120 cc of heparinized blood (Arthrex ACP^®^ double syringe system, Naples, FL, USA) was applied at the fascial level.

#### 2.2.2. Groups 2 and 3: Suture Tape Closure with Biologic Adjuncts

**Figure 2:**
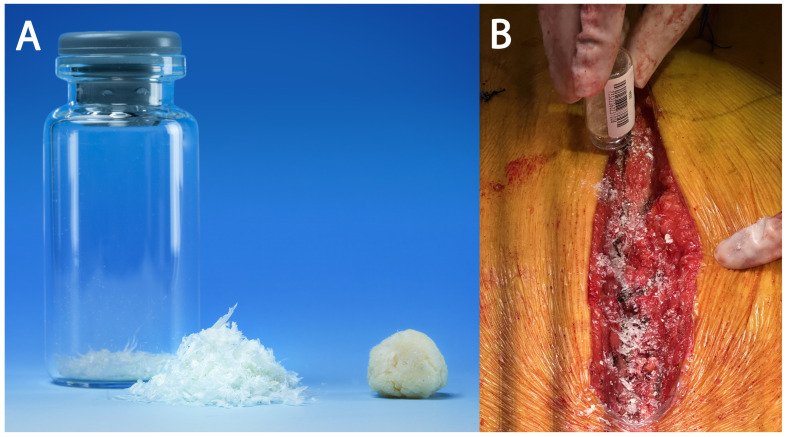
**The aACPA used in this study.** (**A**) The aACPA before use (image used with permission from MTF Biologics). (**B**) Intraoperative image showing the application of the aACPA into the surgical wound.

Ultra-high-molecular-weight polyethylene suture tapes (FiberTape/TigerTape, Arthrex Inc., Naples, FL, USA: Figure 1B) and a tensioner (Figure 1C) were used for sternal reapproximation, as previously described [11]). After wound lavage, 160 mg of aACPA (Salera^®^, MTF Biologics, Edison, NJ, USA) was applied to the sternum and subcutaneous tissues before closure (Figure 2). aACPA was aseptically processed with no terminal irradiation; amnion–chorion membranes were minimally processed to retain the structural properties of the extracellular matrix (ECM). The resulting dehydrated allograft was placed within the surgical wound as a wound covering. Group 2 also received autologous PRP as in Group 1.

### 2.3. Follow-Up and Outcomes

The total ICU and hospital length of stay (LOS) were recorded, as well as hospital mortality, sternal wound infection, and dehiscence. Following our institutional protocol, patients underwent follow-up evaluations at 14 and 30 days postoperatively. Each follow-up visit included a thorough wound inspection and pain assessment using a Numeric Rating Scale (NRS-11) as part of the Universal Pain Assessment Tool (UPAT) [26]. On the NRS-11, patients self-report pain on an 11-point scale (0 to 10), where 0 represents no pain at all and 10 represents the most severe pain requiring bed rest. This scale is based on the patient’s ability to perform activities of daily living (ADLs) and was therefore considered the most useful measure of postoperative pain severity and its impact on functional status. Postoperative pain was deemed significant if the pain score exceeded 4 (significant interference with ADLs). Appropriate analgesia was ensured for all patients with significant pain at follow-up, potentially commencing a course of new opioid medication if required.

### 2.4. Statistical Analysis

The normality of continuous variables was assessed using the Shapiro–Wilk test. Normally distributed data were presented as mean ± standard deviation (SD), and ANOVA was used to compare groups. Non-parametric data were presented as median and interquartile range, with Kruskal–Wallis tests used to compare groups. Categorical variables were presented as N (%) with groups compared using the Chi-square test or Fisher’s test if the expected frequency was less than five. All statistical analyses were undertaken using R v4.4.2 (R Foundation, Vienna, Austria) [27], with a significance threshold of 0.05 used, as is conventional.

## 3. Results

### 3.1. Patient Characteristics

The study population consisted of 365 patients with various risk factors for sternal complications following cardiac surgery. The prevalence of risk factors for sternal complications was similar across the three study cohorts (Table 1). Obesity was the most common risk factor, present in 244 individuals (66.8%). The mean BMI of the study population was 32.37 kg/m^2^. Diabetes mellitus, frailty, and smoking history were also common risk factors. The three groups were also similar when considering other preoperative characteristics (Table 2). The mean age of the total study population was 67 years, with a male predominance (266 patients, 72.9%).

**Figure 3:**
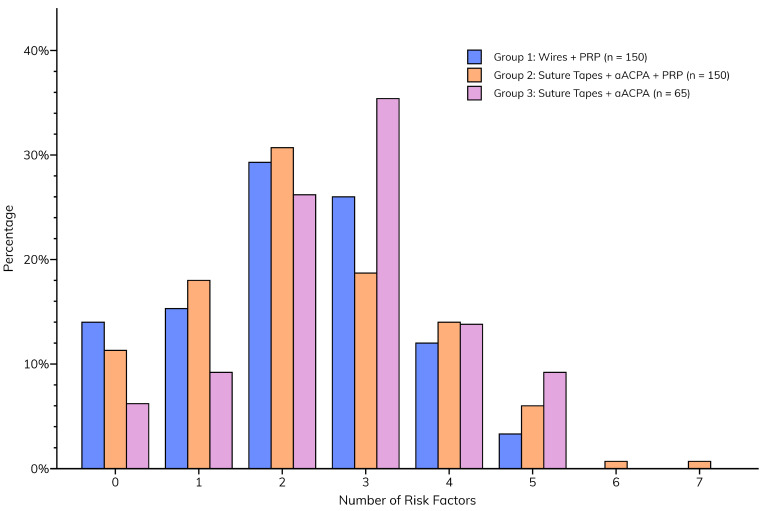
**Total number of sternal risk factors per patient, compared between groups.**

Although the incidences of individual sternal risk factors were similar between groups, the total number of risk factors was significantly higher (*p* = 0.0305) in Group 3 (median = 3 [2–3]) than in Groups 1 and 2 (median for both = 2 [1–3]), implying a greater likelihood of postoperative sternal complications (Figure 3).

### 3.2. Operative Characteristics

The three study cohorts were similar in terms of operative characteristics (Table 3). Most cases (191 patients, 52.3%) involved isolated coronary artery bypass grafting (CABG). Cardiopulmonary bypass, aortic cross-clamp, and total operative times were similar across groups. As is our standard practice, the left internal mammary artery (LIMA) was harvested in a skeletonized fashion in 100% of patients to better maintain sternal perfusion and improve postoperative healing [3].

### 3.3. Postoperative Outcomes

**Figure 4:**
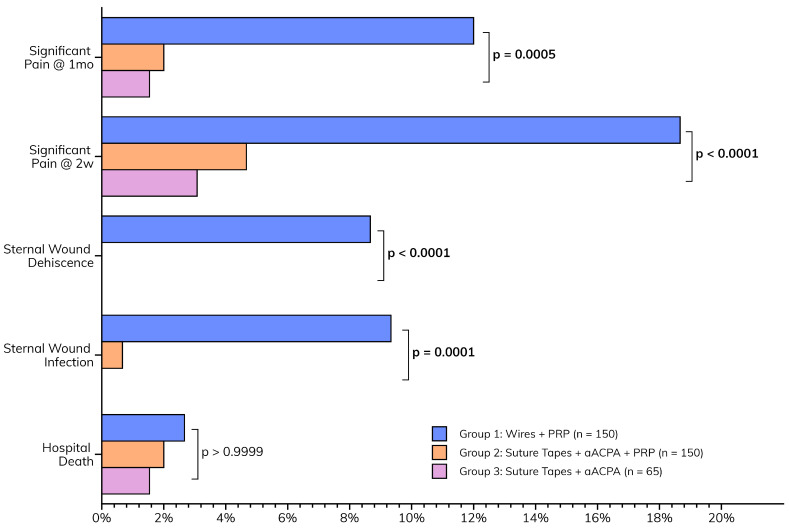
Postoperative outcomes.

The postoperative outcomes collectively suggest that the enhanced wound closure protocol, incorporating aACPA with or without PRP, may be associated with a smoother recovery (Figure 4). This is characterized by the paucity of major sternal complications (Table 4) observed in Groups 2 (suture tapes, aACPA, PRP) and 3 (suture tapes and aACPA without PRP). These included sternal infections (*p* = 0.0001) and dehiscence (*p* < 0.0001), as well as pain at two weeks (*p* < 0.0001) and one month (*p* = 0.0005). This was a significant decrease from the relatively high rates of complications in Group 1 (standard care). Group 3 showed no wound infection or dehiscence, although it was the smallest group. At 2 weeks, only 4.7% of Group 2 and 3.1% of Group 3 patients reported significant pain, compared to 18.7% in Group 1. These trends persisted at 1-month follow-up, further highlighting the success of the protocol.

ICU and hospital stays also fell within reasonable ranges for cardiac surgery patients, with Group 3 achieving the shortest hospital LOS compared to Groups 1 and 2 (Table 4). Although not statistically significant (*p* = 0.2298), this trend supports the notion that optimal wound closure may contribute to a more efficient recovery, potentially facilitating timely discharge.

For outcomes that differed significantly between groups, pairwise comparisons were conducted, with the p-values shown below (Table 5). Outcomes significantly improved with the introduction of our protocol (Group 1 vs. Group 2). The outcomes were similar after revising our protocol to discontinue PRP use (Group 2 vs. Group 3).

## 4. Discussion

This study highlights the evolution and effectiveness of our comprehensive sternal closure strategy in a high-risk cardiac surgery population. Despite similar risk profiles across groups, the introduction of our enhanced wound closure protocol incorporating aACPAs (Groups 2 and 3) significantly improved outcomes compared to traditional wire closure with PRP alone (Group 1).

### 4.1. Key Findings and Implications

Our study cohort of 365 patients represents a high-risk population for sternal complications, with 66.8% being obese (mean BMI 32.37 kg/m^2^), 53.4% frail, and a high prevalence of diabetes mellitus (39.2%) and smoking history (35.9%). Despite these risk factors, sternal infection and dehiscence rates dropped substantially with the enhanced closure protocol, from 9.3% and 8.7% in Group 1 to nearly zero in Groups 2 and 3. Postoperative pain at two weeks and one month also improved significantly, while hospital length of stay showed a trend toward reduction in Group 3. These outcomes were achieved without prolonging operative times, suggesting procedural efficiency and highlighting the limited additional benefits of PRP when combined with aACPA.

### 4.2. Mechanism of Action and Relevance to High-Risk Patients

aACPAs offer unique benefits for wound closure, particularly in high-risk populations with impaired healing capacity, such as those with diabetes, obesity, frailty, or compromised immunity [28]. Through aseptic processing, aACPAs preserve the native extracellular matrix (ECM) components and growth factors, including platelet-derived growth factor (PDGF), vascular endothelial growth factor (VEGF), and transforming growth factor-beta (TGF-β). These have been shown to support angiogenesis, cell proliferation, and tissue remodeling [29]. Additionally, the extracellular matrix (ECM) present in aACPAs may contribute significantly to wound repair. The ECM is a network of proteins, fibroblasts, glycoproteins, and other molecules that provide structural support and facilitate growth [30], acting as a scaffold for cell migration, proliferation, and differentiation [30,31]. This biological scaffold facilitates wound repair even in patients with reduced vascularization, altered immune responses, or increased mechanical stress, as seen in obese and diabetic patients. This allograft also incorporates both amnion and chorion tissues derived from a natural placenta, potentially offering additional advantages over allografts containing solely amnion or chorion. In studies conducted with diabetic mice, aACPAs demonstrated the promotion of granulation tissue formation and accelerated epithelialization of full-thickness wounds compared to allografts containing only amnion membrane [20].

Immunocompromised, diabetic, frail, and obese patients often face challenges in the wound healing process due to various factors, such as impaired circulation, reduced immune function, and compromised cellular activity [32,33,34]. Diabetic patients have up to a fivefold higher risk of developing a postoperative wound infection compared to non-diabetic controls due to altered ECM composition, poor vascularization, and protein imbalance in diabetic wounds [34]. aACPAs may provide a solution to address these concerns, as shown in patients with diabetic foot ulcers [14,15,16,17]. In obese patients, increased adipose deposits can impair wound healing through a range of mechanisms such as reduced vascularization and an altered inflammatory response [32].

This comprehensive approach to wound closure, which simultaneously addresses multiple aspects of tissue repair, may explain the promising results observed in our high-risk patient cohort. By providing a supportive extracellular matrix (ECM) scaffold with preserved native matrix proteins, aACPAs appear to create an optimal environment for sternal wound closure, even in patients with multiple risk factors for complications. Furthermore, the transition from using steel sternal wires to now using suture tapes has also contributed to this improvement in outcomes [11], as they are known to be at least twice as strong as steel while reducing bone cut-through.

### 4.3. Comparison with Previous Research

A 2010 study assessed wound healing over two weeks in mice treated with human placenta extract and demonstrated that, in addition to promoting wound healing through increased levels of TGF-β, the treated mice exhibited faster wound healing compared with controls [35]. Similarly, in vivo examination of processed human placental tissue found that the extracellular matrix sheet demonstrated the development of a full-thickness dermal substitute that closely resembled the cellular organization found in healthy skin, with even new hair follicles and micro-vessels formed [36]. Studies in diabetic mice found that the application of human placental membrane improved wound closure compared to controls, promoted vascularization of the wound bed, increased deposition of granulation tissue, and enhanced closure of the epithelial gap [20]. There was a notable shift toward an anti-inflammatory macrophage phenotype during the wound healing process.

In a clinical setting, Tacktill and colleagues found significant benefits along with reduced surgical complications by using human amnion–chorion allografts in patients undergoing lower extremity reconstructive surgery [13]. Significant improvements in pain, function, and alignment at one-year follow-up were seen, accompanied by reduced surgical complications, such as a lack of wound dehiscence. These positive results are crucial for diabetic patients, who face challenges in wound closure, highlighting the potential benefits of using these allografts to support wound closure. The present study applies these findings to the cardiac surgical field, suggesting that the benefits of aACPA use may extend to wound closure across various surgical specialties. It is also important to acknowledge the contribution that the suture tapes made to the improvement in sternal outcomes. Our group has previously shown a significant improvement by switching from sternal wires, not only in terms of sternal wound infection and dehiscence but also in pain at follow-up [11].

### 4.4. Health Economics Considerations

Preliminary health economics analyses indicate that our enhanced wound closure protocol incorporating suture tapes and aACPA demonstrates promising cost-effectiveness. The Group 3 protocol not only yielded superior clinical outcomes but also reduced costs by eliminating the need for auxiliary products such as sternal support vests, negative pressure dressings, and platelet-rich plasma therapy (PRP), the latter having shown no demonstrable therapeutic benefit. Furthermore, Groups 2 and 3 showed reduced complications and postoperative pain scores compared to Group 1, potentially leading to shortened hospital stays and associated cost savings. While a comprehensive health economics analysis is still in progress, initial work suggests that our enhanced closure protocol offers substantial economic advantages alongside its documented clinical benefits.

### 4.5. Limitations of This Study

While our results are encouraging, it is important to note that our study has certain limitations. As a single-center, retrospective analysis, it may have inherent biases and limited generalizability. However, we hope that this concern is mitigated by the well-matched study groups. A longer duration of follow-up is necessary to comprehensively assess the durability of the outcomes. A formal health economic analysis comparing the three techniques for sternal closure, including any potential savings from reduced complications and shorter hospital stays, would be valuable for healthcare systems considering this technology.

## 5. Conclusions

Cardiac surgery patients with increasingly complex conditions, such as the elderly, frail, immunocompromised, and obese, face significant risks of complications during sternal wound closure, leading to a 20% readmission rate within 30 days. This study aimed to evaluate the use of aseptically processed, non-terminally irradiated amnion–chorion placenta allografts (aACPAs) for median sternotomy wound closure in high-risk patients. The results demonstrate the absence of sternal wound infections, dehiscence, or substantial pain at 14- and 30-day follow-ups, even in the high-risk cohort that typically experiences and reports complications. While promising in reducing postoperative complications, larger studies with extended follow-ups are necessary to comprehensively confirm the efficacy and safety of aACPAs in cardiac surgery sternal wound closure.

## Figures and Tables

**Table 1:** **Relevant risk factors for postoperative sternal complications**. Groups were similar in terms of risk for sternal complications. BMI was compared using ANOVA, and the STS DSWI scores were compared using the Kruskal–Wallis test. All other risk factors were categorical and were therefore compared using the Chi-square test.

Variable	Group 1	Group 2	Group 3	*p*-Value
Number	150	150	65	N/A
BMI (kg/m^2^)	33.31 ± 9.86	31.27 ± 8.69	32.53 ± 4.27	0.2502
STS DSWI Score	0.14 (0.08–0.26)	0.18 (0.08–0.37)	0.20 (0.10–0.26)	0.1834
Obesity	96 (64.0%)	99 (66.0%)	49 (75.4%)	0.2547
Frailty	82 (54.7%)	71 (47.3%)	42 (64.6%)	0.0608
Diabetes Mellitus	59 (39.3%)	56 (37.3%)	28 (43.1%)	0.7296
Previous Sternotomy	9 (6.0%)	11 (7.3%)	7 (10.8%)	0.4706
Smoking History	43 (28.7%)	60 (40.0%)	28 (43.1%)	0.0507
COPD	25 (16.7%)	33 (22.0%)	15 (23.1%)	0.4063
Immunosuppressive Medications	11 (7.3%)	15 (10.0%)	6 (9.2%)	0.7089

**Table 2:** **Other preoperative characteristics and comorbidities**. Groups were similar in terms of overall risk profile. Age was compared using ANOVA. The STS, CHA_2_DS_2_-VASc, and HAS-BLED scores were compared using the Kruskal–Wallis test. All other variables were categorical and were therefore compared using the Chi-square test.

Variable	Group 1	Group 2	Group 3	*p*-Value
Number	150	150	65	N/A
Age (y)	67 ± 11	66 ± 11	67 ± 15	0.7104
Sex: Male	110 (73.3%)	109 (72.7%)	47 (72.3%)	0.9852
STS Score	1.53 (0.73–2.52)	1.48 (0.75–3.02)	1.68 (0.94–3.20)	0.3922
Hypertension	113 (75.3%)	124 (82.7%)	53 (81.5%)	0.2617
Hyperlipidemia	113 (75.3%)	112 (74.7%)	49 (75.4%)	0.9890
Coronary Artery Disease	86 (57.3%)	88 (58.7%)	37 (56.9%)	0.9608
Obstructive Sleep Apnea	37 (24.7%)	41 (27.3%)	23 (35.4%)	0.2702
Heart Failure	24 (16.0%)	31 (20.7%)	15 (23.1%)	0.4007
Chronic Kidney Disease	32 (21.3%)	34 (22.7%)	16 (24.6%)	0.8666
Prior Atrial Fibrillation	24 (16.0%)	17 (11.3%)	11 (16.9%)	0.4064
Prior MI	31 (20.7%)	24 (16.0%)	15 (23.1%)	0.4007
Prior PCI	25 (16.7%)	27 (18.0%)	14 (21.5%)	0.6950
Prior Stroke	6 (4.0%)	8 (5.3%)	4 (6.2%)	0.7647
CHA_2_DS_2_-VASc	3.00 (2.00–4.00)	3.00 (1.00–3.00)	2.00 (1.00–3.00)	0.4700
HAS-BLED	2 (2.00–3.00)	2.50 (2.00–3.75)	2.00 (1.00–2.75)	0.0950

**Table 3:** **Comparison of operative characteristics**. Groups were similar considering operative characteristics. Cardiopulmonary bypass, aortic cross-clamp, and operative times were compared using ANOVA. All other variables were categorical and were therefore compared using the Chi-square test.

Variable	Group 1	Group 2	Group 3	*p*-Value
Number	150	150	65	N/A
Elective	121 (80.7%)	124 (82.7%)	58 (89.2%)	0.3041
Procedure Type				0.8339
CABG ± Maze	80 (53.3%)	76 (50.7%)	35 (53.8%)	
Valve ± Maze	37 (24.7%)	48 (32%)	18 (27.7%)	
CABG & Valve ± Maze	16 (10.7%)	13 (8.7%)	7 (10.8%)	
Aortic	17 (11.3%)	13 (8.7%)	5 (7.7%)	
LIMA Skeletonization	90 (100%)	83 (100%)	39 (100%)	>0.9999
Cardiopulmonary Bypass (min)	93 ± 35	89 ± 38	90 ± 29	0.3922
Aortic Cross-Clamp (min)	66 ± 32	68 ± 26	67 ± 23	0.7808
Operative Time (min)	221 ± 41	226 ± 46	224 ± 48	0.4751

**Table 4:** **Comparison of postoperative outcomes between study groups**. These were significantly better in Groups 2 and 3 than in Group 1, specifically, the rates of sternal wound infection and dehiscence, as well as the incidence of postoperative incisional pain at follow-up. The ICU and total hospital LOS were compared using the Kruskal–Wallis test. All other outcomes were categorical and were therefore compared using the Chi-square test.

Variable	Group 1	Group 2	Group 3	*p*-Value
Number	150	150	65	N/A
ICU LOS (d)	3.00 (2.00–5.00)	2.50 (2.00–4.50)	3.00 (2.00–4.00)	0.9212
Hospital LOS (d)	8.00 (6.00–11.00)	7.00 (5.00–11.00)	6.00 (5.25–9.00)	0.2298
Hospital Death	4 (2.7%)	3 (2.0%)	1 (1.5%)	>0.9999
Sternal Wound Infection	14 (9.3%)	1 (0.7%)	0 (0.0%)	0.0001
Sternal Dehiscence	13 (8.7%)	0 (0.0%)	0 (0.0%)	<0.0001
Significant Pain: 2 w	28 (18.7%)	7 (4.7%)	2 (3.1%)	<0.0001
Significant Pain: 1 mo	18 (12.0%)	3 (2.0%)	1 (1.5%)	0.0005

**Table 5:** **Pairwise comparisons of significantly different outcomes**. These were obtained using pairwise Fisher tests.

Variable	Group 1 vs. 2	Group 1 vs. 3	Group 2 vs. 3
Sternal Wound Infection	0.0007	0.0066	>0.9999
Sternal Dehiscence	0.0002	0.0111	N/A
Significant Pain: 2 w	0.0002	0.0021	0.7264
Significant Pain: 1 mo	0.0010	0.0159	>0.9999

## Data Availability

All data will be made available upon reasonable request to Dr. Zain Khalpey (zain@khalpey.ai).
